# Predicting the Linear Low-Density Polyethylene Content of Custom Polypropylene Blends and Post-Consumer Materials Using Rheological Measurements

**DOI:** 10.3390/polym16223169

**Published:** 2024-11-14

**Authors:** Dominik Kaineder, Christian Marschik, Ingrid Trofin, Sabine Hild

**Affiliations:** 1 Competence Center CHASE GmbH, Hafenstraße 47-51, 4020 Linz, Austria; christian.marschik@chasecenter.at; 2 Institute of Polymer Science, Johannes Kepler University, Altenbergerstraße 69, 4040 Linz, Austria; ingrid.trofin@jku.at (I.T.); sabine.hild@jku.at (S.H.)

**Keywords:** characterization, post-consumer polypropylene, polyolefin, blend, contamination

## Abstract

Contributing to a sustainable economy requires the use of pure recycled materials. Analyzing polyolefin post-consumer materials and cross-contaminations in these materials is an essential part in ensuring consistent product quality. Therefore, the aim of this work was to quantify the linear low-density polyethylene (LLDPE) content in polypropylene (PP)-dominant strips. The materials investigated included virgin PP, custom PP-LLDPE blends and PP post-consumer recyclates. To this end, differential scanning calorimetry (DSC) and parallel-plate rheometry were used. For complementary measurements, Raman spectroscopy and atomic force microscopy (AFM) were employed, confirming the morphological occurrence of LLDPE enclosed in PP up to 30 wt%. The DSC measurements demonstrated that the evaluated specific melt and recrystallization enthalpies alone are insufficient to quantify the LLDPE content, especially at 1–10 wt%. The rheometric results showed a strong correlation between the cross-over point (COP) and zero-shear viscosity for pure PP grades, and there was a deviation from this correlation depending on the LLDPE content in the PP-LLDPE blends. An approach for determining low (1–15 wt%) and medium (up to 30 wt%) LLDPE quantities in PP via two mathematical models is proposed based on the rheometric measurements of custom blends and can be applied to assess the level of LLDPE contamination in PP post-consumer materials.

## 1. Introduction

The recycling of polymer waste to obtain new polymer products is a key approach to reducing environmental pollution and the overuse of natural resources such as oil and gas. In the European Union (EU), the plastic subgroup that makes up the largest proportion (~50%) in terms of overall consumption is formed by polyolefins: polypropylene (PP) and polyethylene (PE) [1,2]. Techniques for mechanically recycling PP and PE are thus key approaches in reaching the goal of 55% plastic recycling by 2030, as set by the EU [3]. However, any form of recovery of polyolefins poses challenges. In mechanical recycling, process-related molecular-chain degradation caused by multiple production processes results in recycled PP with a lower viscosity compared to its virgin form. With PE, in contrast, process-related “degradation” results in the crosslinking of molecular chains [4,5] and—depending on the degree of crosslinking—a higher viscosity, mainly in the low-shear domain [6].

Since PP and any variant of PE (high-density, low-density and linear low-density polyethylene (LLDPE)) have similar physical properties (e.g., density, characteristic temperatures and polarity) and application areas (e.g., packaging) [7], polyolefins are difficult to separate during waste processing. This leads to the inevitable contamination of PP recyclates with PE and vice versa [8,9], which results in conflicting behaviors concerning viscosity when process-related chain scission takes effect. The various shear loads during the recyclate extrusion process and process-related degradation lead to PP domains with a lower viscosity and further crosslinking of any PE contaminants. As virgin polyolefins are typically purchased according to a specified product performance, the initial industrial assessment on which the acceptance of incoming PP and PE feedstocks is based is often limited to Melt Flow Rate (MFR) measurements. This single-point measurement only considers the viscosity at one shear rate to characterize virgin materials. However, PE that cross-contaminates PP can significantly alter the relationship between viscosity and shear rate. PE-contaminated post-consumer PPs with MFRs identical to that of their virgin counterparts will not exhibit the same behaviors in production processing. This may result in suboptimal product processing conditions and localized blemishes. Therefore, the proper analysis of the purity of incoming feedstock materials is crucial when producing high-performance products. To achieve the optimum representative value of blend or post-consumer recyclate samples, industrial-style production facilities are often used to simulate and provoke effects in a realistic production environment for the material used. While the inorganic content can often be quantified accurately by thermo-destructive and spectroscopic methods, polyolefin cross-contaminations, especially at a low content (<15 wt%), require a different approach due to their physical similarities. Thermal, rheometric and micro- and spectroscopic methods are commonly used to characterize polyolefin recyclates [10,11]. Differential scanning calorimetry (DSC) is a state-of-the-art method that measures the difference in heat flow between a reference and sample, and can therefore be used to characterize the specific melt enthalpy of a crystalline sample (or of the crystalline domains of a semicrystalline polymer) [12]. One might expect DSC experiments to show accurate results, as the melt and recrystallization enthalpies of PP and PE ought to reflect the respective mass fractions, which would allow the weight fractions of the polymers in a blend to be calculated. Camacho and Karlsson [10] reported in 2001 that this quantification was not sufficiently accurate for low quantities below 0.91 wt%. In 2024, Ragaert et al. [13] showed that the presence of a polymer contaminant reduces the degree of crystallization of the majority of the material, and used machine learning for correct quantifications [10,13].

Vibrational spectroscopy techniques, namely IR and Raman spectroscopy, allow for the differentiation between PP and PE and are commonly used as industrial separation techniques in waste management for recycling purposes [10,14]. These methods are also often employed to detect cross-contaminations and to characterize the distribution behavior of PE in PP. However, minuscule inclusions (<1 µm) of cross-contaminating PE in PP may not be detected properly, as the laser spot size of 570 nanometers limits the resolution (which is also the case in confocal Raman spectroscopy). Atomic force microscopy (AFM), in contrast, is capable of quantifying inclusions smaller than 1 µm, but the area it can analyze is limited to the nano- and micrometer ranges [15,16].

A more macroscopic investigation approach is rheometry. Here, oscillatory parallel-plate rheometry was used as a state-of-the-art method to characterize the flow properties of a polymer melt. If a monodisperse, homogeneous melt is analyzed, complex zero-shear viscosity ($\eta_{0}$) and the COP correlate with the molar mass and its distribution. Although none of these correlations apply to blended materials, the effect on the storage modulus and viscosity of cross-contaminating PE in PP in relation to the mass fraction and inclusion size is well documented and has been studied in virgin blends [4]. However, there is little research using plate–plate rheometry on PE contaminating post-consumer PP recyclates. Moreover, the use of a quantitative characterization approach for the mass fractions of LLDPE in PP materials using systematic diversions of known correlating rheological properties is rare.

We therefore formulated the following three hypotheses:

(1) DSC is a suitable method for approximating the quantity of cross-contamination in virgin blends and post-consumer recyclates processed under quasi-industrial conditions.
(2) In-depth micro- and nanometer-scale characterization of PE in PP, including contaminant size and distribution, can be performed using Raman spectroscopy and AFM.
(3) The mass fraction of linear low-density PE in PP (both virgin blends and recyclates) can be quantified with a new approach that combines plate–plate rheometry measurements of zero-shear viscosity and the cross-over point (COP) with mathematical models based on the opposing trends of process-related degradation in PP and PE.

## 2. Materials and Methods

The materials investigated in this work (see Table 1, Table 2 and Table 3) consisted of virgin and recycled isotactic PP and LLDPE. In total, we used ten virgin PP samples, three virgin LLDPE samples, thirteen virgin PP+PE blends and five PP recyclates.

### 2.1. Materials and Sample Manufacturing and Preparation

#### 2.1.1. Sample Manufacturing: Using Extruder “MX” to Produce Strips from Manually Pre-Mixed Granules

**Figure 1:**
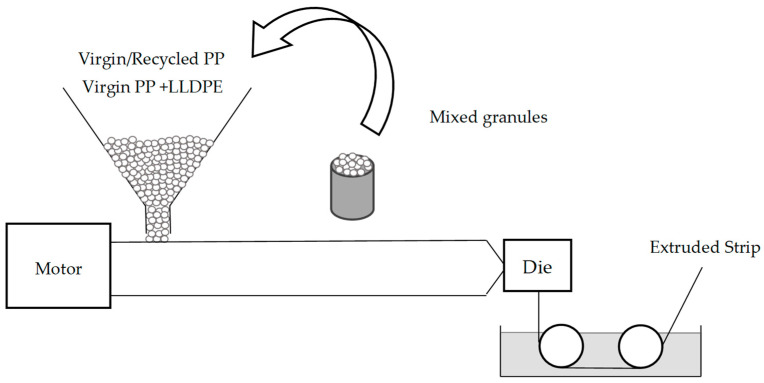
“MX” strip extrusion process using manually prepared granule mixtures.

To observe and compare the effects caused by LLDPE contained in PP in post-consumer recyclates, strips were manufactured from (i) virgin PP and LLDPE (base properties shown in Table 1), (ii) custom PP-PP blends and PP-LLDPE blends, and (iii) recycled post-consumer PP, as listed in Table 2. Figure 1 schematically shows the compounding of pre-mixed granules of these virgin blends or recycled granules. We produced samples in the form of strips, using a 30D single-screw extruder (hereafter referred to as Extruder “MX”) equipped with a slot die with a die cross-section of 200 mm width and 2 mm thickness [17]. The resulting strip was 40–50 mm wide and 0.05–0.4 mm thick and was the result of a caterpillar haul-off at a speed of 5 m/min. The temperatures along the extruders were as follows: (i) feeding zone, 175 °C; (ii) compression zone, 225 °C; (iii) metering zone, 235 °C; and (iv) die, 240 °C. The screw speed was 110 rpm for strip production, which amounts to a throughput of roughly 3.5 kg/h. We used PP 525P, a conventional extrusion grade [18], for strip sample production and for blending with LLDPE. All other materials are listed in Table 2 [19,20,21,22].

**Table 1:** Base properties of virgin materials used [19,20,21,22].

Material Grade	Melt Peak Temperature/°C	Melt Flow Rate ISO 1133 [23]/ g/10 min (2.16 kg)
501P	165.24	0.8
525P	163.98	3.0
HDMO810	162.94	10
Mosten MA350	162.20	50
525P + 50 wt% Mosten MA350	162.47	-
Q1018H	122.40	1.0

**Table 2:** Strips extruded with the “MX” extruder.

Material	Fractions Added wt% PP|wt% LLDPE	Material Grade	Company
Virgin PP/ Virgin PP Blends	100%|0%	501P	Sabic (Riad, Saudi Arabia)
		525P	Sabic
		HD810MO	Borealis (Linz, Austria)
		525P + 5 wt% Mosten MA350	Sabic; Unipetrol (Prag, Czech Republic)
		525P + 10 wt% Mosten MA350	Sabic; Unipetrol
		525P + 50 wt% Mosten MA350	Sabic; Unipetrol
Virgin LLDPE	0%|100%	Q1018H	Lotrène (Doha, Qatar)
Virgin PP + Virgin LLDPE	100%|0%	525P	Sabic
	95%|5%	525P + Q1018H	Sabic; Lotrène
	90%|10%		
	80%|20%		
	75%|25%		
	70%|30%		
Post-Consumer Rec. PP A–C	100%|0%	A	Recycler A
		B	Recycler B
		C-1	Recycler C
		C-2	Recycler C
		C-3	Recycler C

#### 2.1.2. Sample Manufacturing: Using Extruder “LX” to Produce Strips from Granules Pre-Mixed by an Automated Dosing System

**Figure 2:**
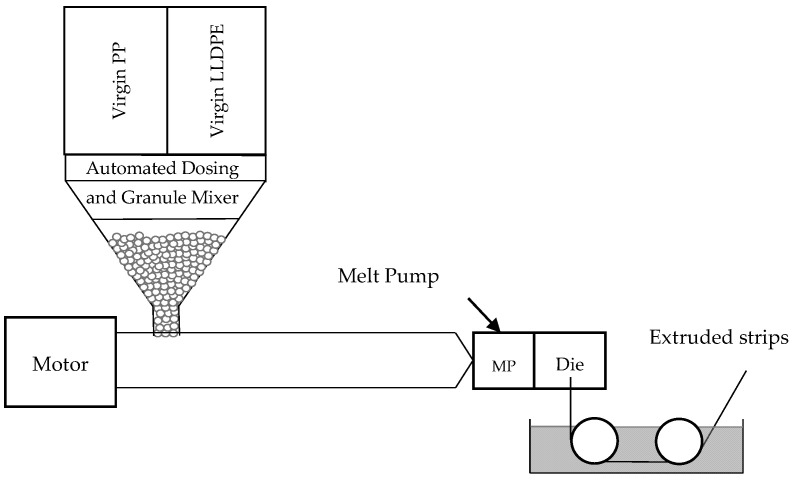
“LX” strip extrusion process using mixtures of granules prepared by an automated dosing system.

The approach of mixing granules manually may be insufficiently precise to obtain very small increments in the LLDPE fraction when simulating contaminated sample strips [24]. Hence, we used an automatic dosing and mixing system that fed material to an extruder (30D; Extruder “LX” in Figure 2) and produced a second set of sample strips that were 25 mm wide and 1 mm thick and had a more precise, reproducible LLDPE content. The temperature profile was similar to that used before: (i) feeding zone, 205 °C; (ii) compression zone, 220 °C; (iii) metering zone, 230 °C; and (iv) die, 240. The screw speed was about 70 rpm to provide the working pressure for the melt pump that supplied a constant flow of 18 kg/h. We assumed that the LLDPE increments from the automated dosing system would be more accurate and give insights into differences in material behavior between the incremental steps manufactured using Extruder “MX” (Table 2), especially at low LLDPE contents. Since only these smaller LLDPE increments were of interest, no additional pure PP or recyclate samples were produced. The proportions of LLDPE contamination in the standard 525P-grade PP produced by the automated dosing system are presented in Table 3.

**Table 3:** Strips extruded with Extruder “LX”.

Material	Fractions Added wt% PP|wt% LLDPE	Material Grade	Company	
Virgin PP + Virgin LLDPE	100%|0%	525P	Sabic	
	99%|1%	525P + Q1018H	Sabic; Lotrène	
	98%|2%			
	97%|3%			
	96%|4%			
	94%|6%			
	92%|8%			
	90%|10%			
	85%|15%			

#### 2.1.3. Sample Preparation for Raman Spectroscopy and AFM

A set of samples was prepared from the extruded strips to enable investigation by AFM and Raman spectroscopy. These methods require a flat strip surface, which—for the samples with various virgin PP-LLDPE ratios and those containing recycled materials—was achieved by melting them between two clean, thin glass slides at 200 °C for 5 min on a Linkam THMS 600 heating stage controlled by a Linkam TMS 93 temperature control unit (Redhill, UK) and then cooling them at 10 °C/min to 144 °C. An isothermal 1 h step at 144 °C was used to allow the PP fraction to crystallize before the LLDPE. This temperature was selected based on DSC measurements, which indicated a PP melting temperature of approx. 164 °C. The samples were then cooled to ambient temperature. In order to more clearly distinguish between the two polymers, additional virgin PP-LLDPE strips were melted and then directly cooled to ambient temperature.

### 2.2. Methods and Experiments

#### 2.2.1. Rheometry

Rheometric experiments were conducted using a plate–plate rheometer (MCR 502; Anton Paar; Graz, Austria). The measurement system consisted of two parallel plates with a mobile plate that was 25 mm in diameter. For the experiments at 200 °C and with a measuring gap of 0.8 mm, we used standard amplitude and frequency sweeps ranging from 0.01 to 5% deformation at 150 rad/s and from 150 to 0.01 rad/s at 5% deformation [6]. The 5% deformation value was chosen since the moduli during the amplitude sweep showed no indication of a divergence into the nonlinear viscoelastic area. MX-produced strips were stacked to achieve a thickness > 0.8 mm using the PP25 measurement system (Anton Paar; Graz, Austria). LX-produced strips already had the appropriate dimensions and required no stacking before the rheometric analysis. The rheo-compass software (Version 1.31.43) was used to (i) evaluate the storage and loss modulus intersection (i.e., cross-over point) and (ii) extrapolate the viscosity using the Carreau–Yasuda regression function [25].

#### 2.2.2. Differential Scanning Calorimetry

Strip samples punched out from the PP-LLDPE variants were used for DSC (DSC3+, METTLER; Vienna, Austria) analysis. The measurement program comprised two heating steps and one cooling step ranging from 25 to 250 °C at 10 °C/min, with isothermal steps lasting three minutes to ensure thorough heating of the sample after each dynamic increment. The crucible used was a 40 µL aluminum pan with a punctured lid. The whole measurement was conducted with nitrogen as purging gas. Melt and recrystallization enthalpies were evaluated with the StarE V18.00b software.

#### 2.2.3. Atomic Force Microscopy

All AFM measurements were performed in air and at room temperature under ambient conditions, using the dynamic mode on an MFP-3D stand-alone device from Asylum Research, Oxford Instruments. Si-cantilevers with an aluminum reflex coating from Budget Sensors were used, with a spring constant of 48 N/m. Imaging was performed at 5% below the resonant frequency of the probes. An excitation amplitude of 5 V was used for tuning, and the measurements were carried out at set points between 55% and 70% of this value. Both height images and phase images (obtained simultaneously) were recorded at a scan rate of 0.5 Hz with a 512 × 512 resolution. All images were displayed and processed using the Gwyddion V2.65 software (free and open-source software covered by a GNU General Public License).

#### 2.2.4. Raman Spectroscopy

A confocal Raman spectroscope (WiTec alpha300; Oxford Instruments; Abingdon, UK) with a laser with an excitation wavelength of 532 nm and power of 5 and 15 mW was used to investigate the contaminants identified by AFM. The method employed was an “image scan”, covering areas with dimensions of 14 µm × 14 µm and 10 µm × 10 µm at a resolution of 250 nm (56 spectra along 56 lines; 40 spectra along 40 lines) and with an integration time of 0.5 s. In this “image scan” mode, the spectrometer systematically scanned across the surface of a sample to collect Raman spectra at every 250 nanometers, generating a two-dimensional chemical map. Each spectrum captured displays a spectrometric fingerprint of the scanned surface, providing insights into the composition and distribution of the macromolecules present. Screening for a fingerprint band of a component results in an image displaying the highest relative intensity of that peak as bright colors and the lowest measured intensity as dark shades. A “×100” objective with a numerical aperture of 0.9 (Nikon; Tokyo, Japan) was used and the “Project Five” V5.3 software was employed to evaluate the spectra.

#### 2.2.5. Experiments

The samples and the corresponding analysis methods are listed in Table 4 and Table 5. Every sample was investigated by rheometry, since this was the main focus of this work. DSC was performed to quantify the LLDPE content in the custom blends. Raman spectroscopy and AFM served as complementary methods to qualitatively identify LLDPE inclusions in the PP.

## 3. Results

The results of this work are structured into three parts. The characterization part demonstrates that the LLDPE contents of both sets of samples cannot be quantitatively assessed by DSC without corrections [13]. The investigative part displays the AFM and Raman measurements that complement the DSC method results and presents a small-scale analysis of the LLDPE inclusions in the PP. The third part provides the results of the rheometric testing as an alternative characterization method.

### 3.1. PP-LLDPE Content Investigation by DSC

**Figure 3:**
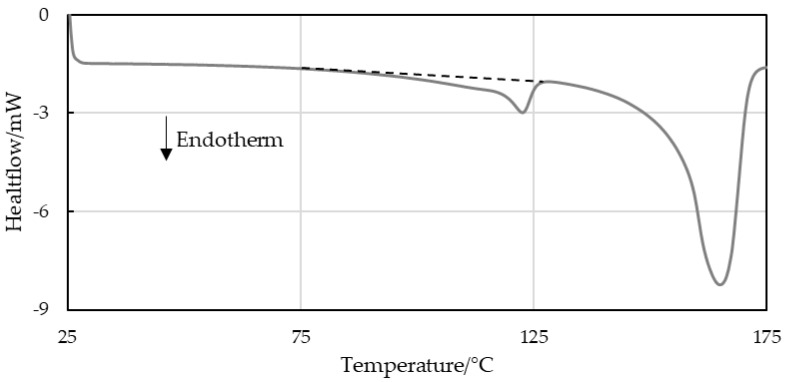
Schematic linear baseline integration of the melt enthalpy area of LLDPE enclosed in PP.

**Figure 4:**
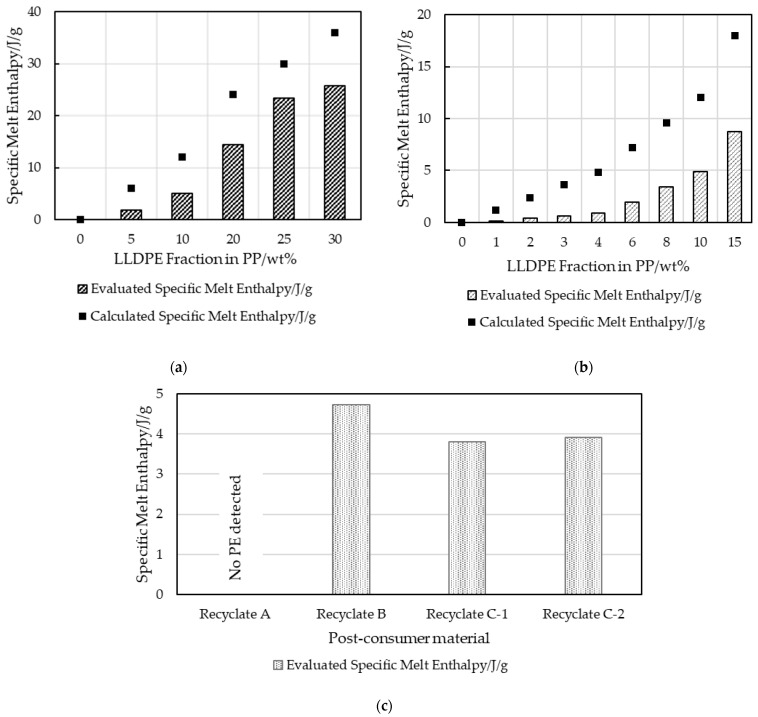
Calculated and evaluated (using linear baseline method) specific melt enthalpies of crystalline LLDPE inclusions in virgin blended (**a**) MX strips and (**b**) LX strips, and specific melt enthalpies for (**c**) recyclate MX strips with unknown LLDPE content.

DSC was used to evaluate the melt enthalpies of the second heating step of the investigated materials. The specific “calculated” melt enthalpies were determined via the known crystallizable LLDPE fractions added to the custom blends based on a pure LLDPE sample which had a result of 120 J/g (5% LLDPE peak in 95% PP resulted in a calculated melt enthalpy of 6 J/g). The “evaluated” specific melt enthalpy was determined using a line integration method. This linear baseline evaluation is shown schematically in Figure 3. Figure 4 plots the evaluated and calculated specific melt enthalpies of LLDPE in virgin PP-LLDPE blends. Although the enthalpies obtained by the linear baseline evaluation exhibited a continuous increase with increasing LLDPE content, they did not match the calculated enthalpies, especially at low LLDPE concentrations. Since the LLDPE content of the post-consumer strips was unknown, the melt enthalpies could not be calculated.

The factors that complicated the evaluation included overlapping LLDPE and PP melt areas and inconsistent baseline limits for peak integration [13]. While virgin LLDPE began to melt at 30 °C, overlapping melt domains made it impossible to quantify the LLDPE fraction. The linear baseline evaluation of the calculated enthalpy of LLDPE in comparison to its actual integration area value is shown in Figure 4.

**Figure 5:**
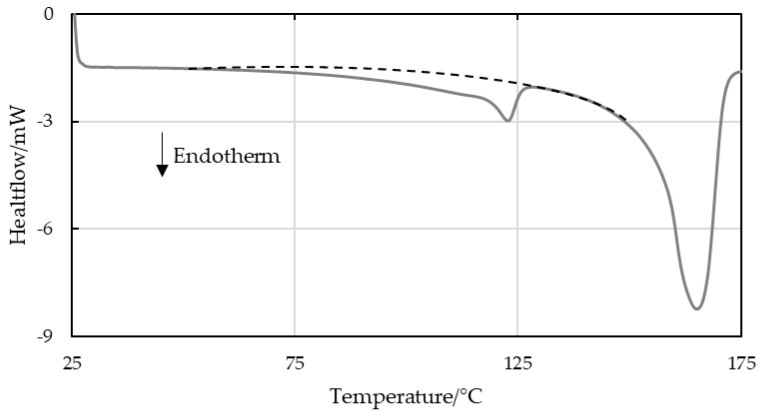
Schematic spline baseline integration of the melt enthalpy area of LLDPE enclosed in PP.

**Figure 6:**
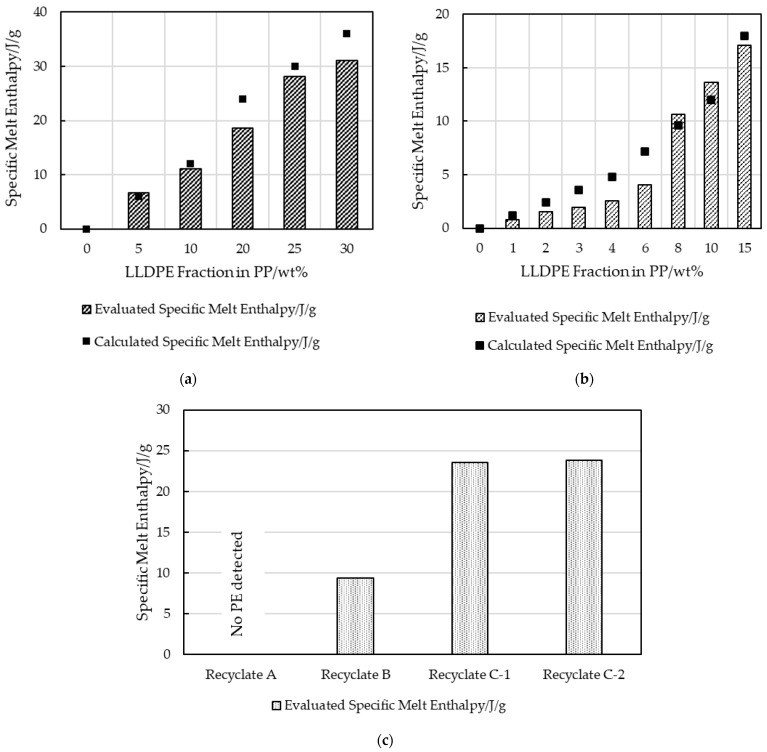
Calculated and evaluated (using spline baseline integration) specific melt enthalpies of crystalline LLDPE inclusions in virgin blends for (**a**) MX strips and (**b**) LX strips, and specific melt enthalpies for (**c**) recyclate MX strips with unknown LLDPE content.

The data were also evaluated using spline baseline integration (Figure 5). This considers the curvature of the baseline but depends significantly on the integration limits chosen. Again, the calculated and evaluated melt enthalpies did not match entirely, as can be seen in Figure 6 for strips with an LLDPE content of up to 30 wt%.

Additionally, the linear rule of mixing for crystallinity of two different materials was applied [26]. For a theoretical crystallinity of 100%, industrial-grade LLDPE has a melt enthalpy of 297 J/g; however, under real-world conditions, crystallinity is typically 40% with a melt enthalpy of 120 J/g [11,13]. The linear rule of mixing based on weight fractions (Equation (1), [26]) dictates that an intermediate recrystallization peak area integral value between the two extrema would be obtained (see Equation (2)). Since the true recrystallization enthalpies of LLDPE and PP virgin materials are known from experiments with pure samples, the linear rule of mixing can be implemented. The evaluated recrystallization enthalpies for the 525P PP amounted to 99 J/g in the LX strips and to 105 J/g in the MX strips, while the Q1018H LLDPE enthalpy was 120 J/g in its pure unblended state. These values agree with the literature standards for PP, with an upper limit of 207 J/g at 100% crystallinity and a value of 105 J/g that is commonly measured in extrusion-grade material under real conditions [7].

(1) $${\Delta H}_{A}\cdot {wt}_{A}\%+{\Delta H}_{B}\cdot {wt}_{B}\%={\Delta H}_{Mix}$$

(2) $$99\frac{J}{g}|105\frac{J}{g}\cdot {wt}_{PP}\%+120\frac{J}{g}\cdot {wt}_{LLDPE}\%={\Delta H}_{PP/LLDPE\;Mix}\;$$

**Figure 7:**
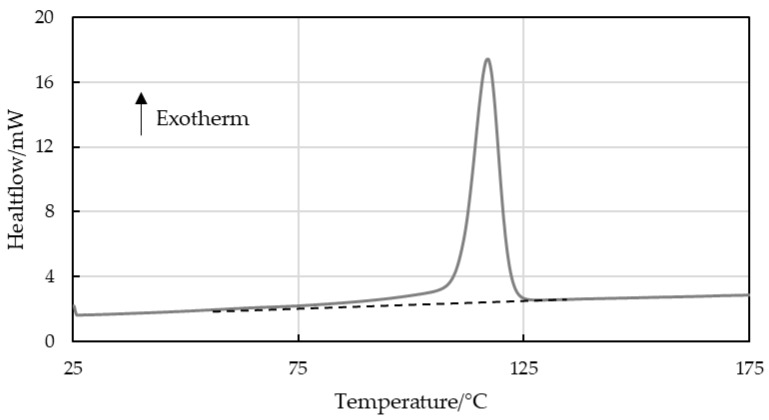
Schematic baseline integration of a combined PP/LLDPE recrystallization enthalpy area.

**Figure 8:**
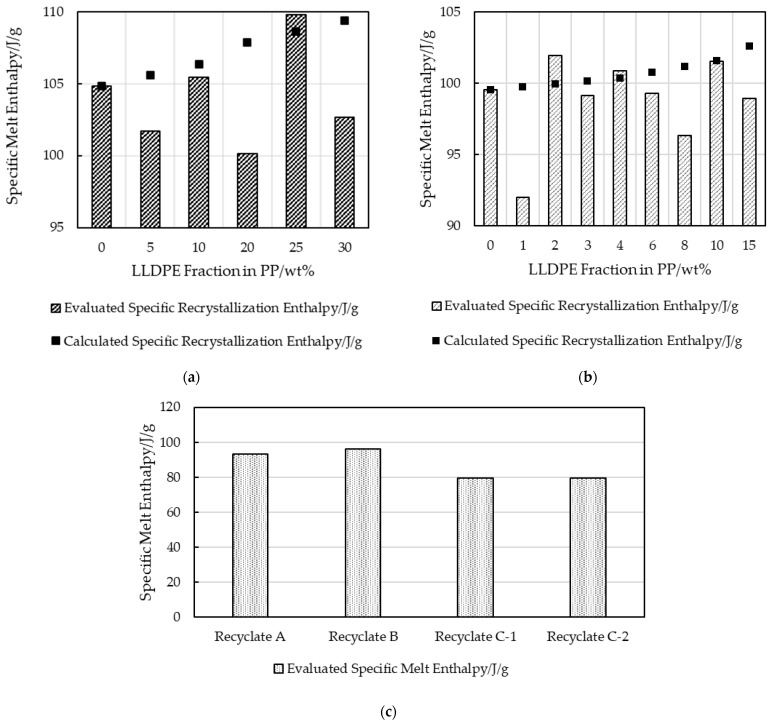
Calculated and evaluated recrystallization enthalpies of extruded virgin blends for (**a**) MX strips and (**b**) LX strips; and recrystallization enthalpy of (**c**) post-consumer strips with unknown LLDPE content.

Figure 7 and Figure 8 show that the recrystallization enthalpies expected for virgin PP and LLDPE were not obtained for the virgin blends nor for the recyclate samples. The LLDPE content in the recycled grades could therefore not be estimated, but, qualitatively, the presence of LLDPE was confirmed.

### 3.2. Complementary AFM and Raman Spectroscopy Measurements

**Figure 9:**
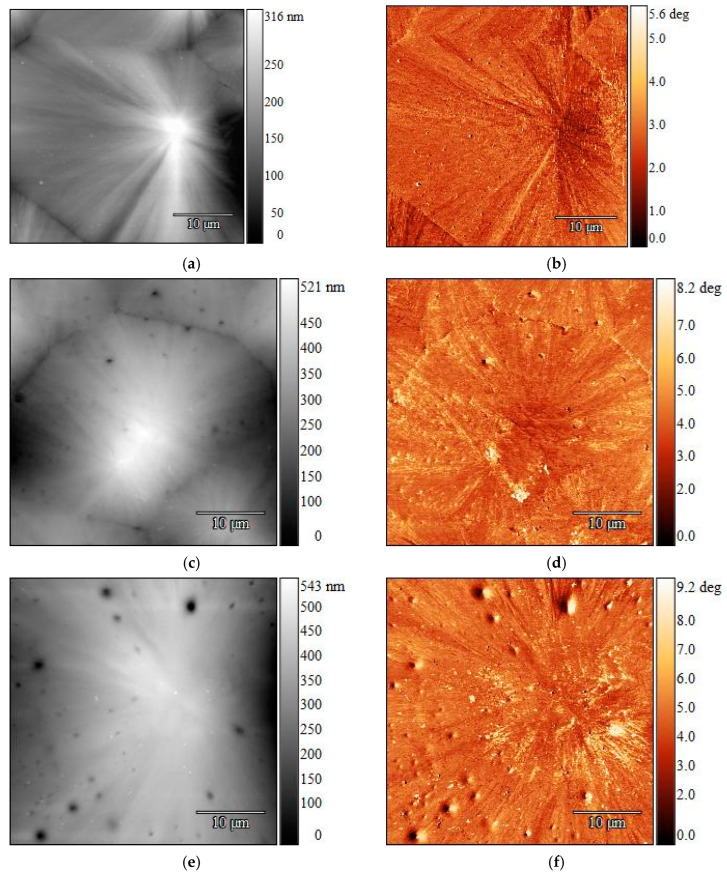
AFM height images (**left**) and corresponding phase images (**right**) of MX strips of pure PP (**a**,**b**) and virgin PP-LLDPE blends with various polymer ratios (5 wt% LLDPE in (**c**,**d**) and 10 wt% LLDPE in (**e**,**f**)) after melt crystallization with an isothermal step. PP spherulites can be seen in all three cases. The presence of LLDPE was confirmed by the appearance of darker spots after melt crystallization (**c**,**f**), where a higher LLDPE content led to more spots. Size scan: 40 × 40 µm.

**Figure 10:**
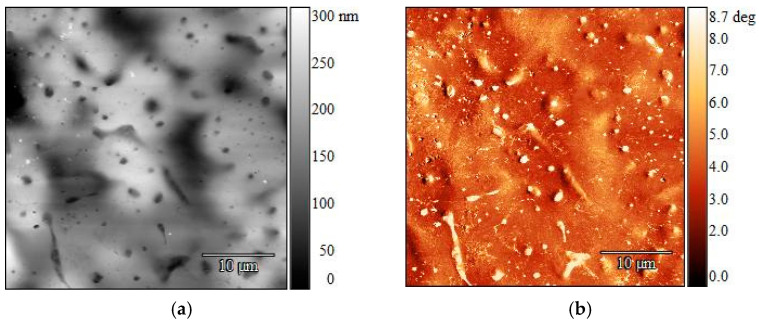
AFM height images (**left**) and corresponding phase images (**right**) of PP-LLDPE MX strips with various polymer ratios (25 wt% LLDPE in (**a**,**b**)) after melt crystallization with an isothermal step. Spherulites could not be discerned with an LLDPE content greater than 25 wt%. An increase in LLDPE content led to an increase in the size of the domains. Size scan: 40 × 40 µm.

Further AFM investigations at high resolution showed a purely homogeneous morphology in the virgin samples and heterogeneous structures in the contaminated samples. The isothermal step at 144 °C during cooling caused PP spherulites of various sizes to form (at the centers of the samples) in the MX strips, as can be seen in Figure 9a,b. Figure 9 displays the spherulites formed towards the middle of the samples in strips of pure PP (Figure 9a,b) and in virgin PP-LLDPE strips with 5 wt% LLDPE (Figure 9c,d) and 10 wt% LLDPE (Figure 9e,f). With increasing LLDPE content, an increasing number of darker, round spots can be seen on the sample surfaces. Their presence may be due to the contraction of the LLDPE domains during crystallization [27], which takes place at a lower temperature than for PP [28,29]. Additionally, due to a slight difference in density between the two polymers in solid form, 0.905 g/cm^3^ for PP and 0.918 g/cm^3^ for LLDPE, it could be assumed that these inclusions ranging from microscopic to nanoscale sizes are LLDPE domains.

The darker spots in the AFM topographic images correspond to lower heights [30]. An increase in the sample height was observed with an increase in the LLDPE weight percentage, starting at a maximum height of 316 nm in the virgin PP and going up to 543 nm with a 10 wt% LLDPE content. However, for the samples obtained with an isothermal step during crystallization, the two polymers could not be clearly distinguished in the phase images obtained since topography had a strong influence on phase contrast in this case. With a further increase in the LLDPE fraction, spherulites could no longer be discerned, but expansion of the LLDPE domains was observed, as illustrated by Figure 10 for 25 wt% (Figure 10a,b).

**Figure 11:**
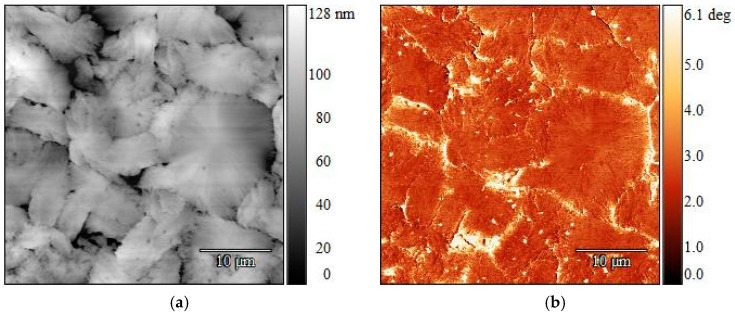

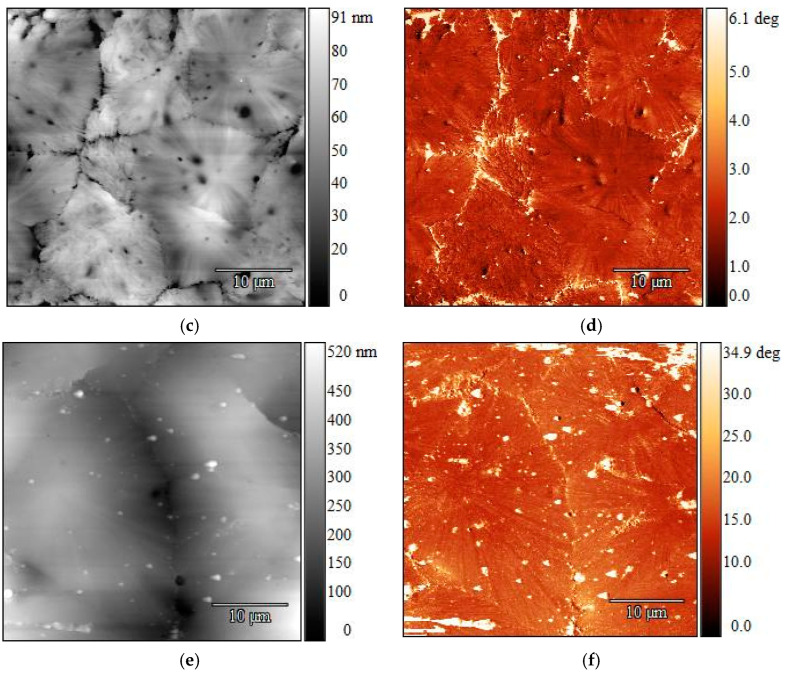
AFM height images (**left**) and corresponding phase images (**right**) of post-consumer recyclate A (**a**–**d**) and recyclate B (**e**,**f**) after melt crystallization with an isothermal step. Spherulites were unevenly distributed through the surface. Presence of LLDPE was confirmed by the appearance of darker spots after melt crystallization (**c**,**d**), as was observed for the virgin materials that were previously tested. Size scan: 40 × 40 µm.

In both recycled materials that were tested, spherulites of various sizes could be observed, spread unevenly through the strip surfaces (Figure 11). Recyclate A followed the trend of virgin PP, forming a few larger spherulites across the sample. The lack of darker, round spots after melt crystallization for the post-consumer materials confirmed the high purity of the recycled PP used (Figure 11a,b). AFM indicated the presence of LLDPE in recyclate A, which was undetected by DSC. At 10 wt% LLDPE (Figure 11c,d), the post-consumer strip behaved as if it contained virgin polymers, exhibiting darker, round spots of various sizes. For recyclate B (Figure 11e,f), AFM showed a rough surface and the presence of PE, which was also detected by DSC, although no LLDPE was mixed into it in the experiments. Again, the topography greatly influenced the phase contrast, with LLDPE still being shown as a lower indentation on the surface. Nonetheless, the lighter domains in the phase images could be attributed to the different fillers added during the recycling process.

**Figure 12:**
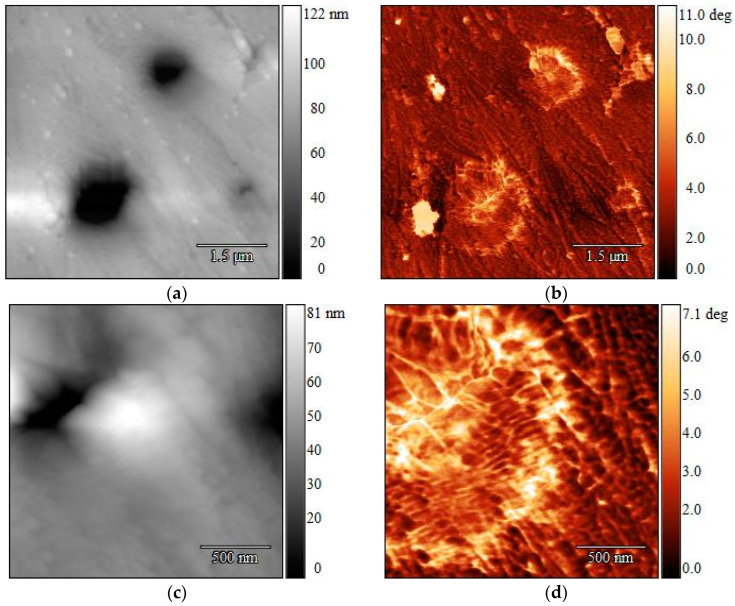
AFM height images (**left**) and corresponding phase images (**right**) of 5 wt% LLDPE in PP MX strips after melt crystallization without an isothermal step. Parallel PP lamellae can be seen in all images (**a**–**d**). Presence of LLDPE in the area of the dark spots was confirmed by the difference in the material in the phase images (**c**,**d**). Size scan: 6 × 6 µm (**top**) and 2 × 2 µm.

The two polymers were more distinguishable when the virgin polymer strips were melted and then rapidly cooled to ambient temperature without an isothermal step: the LLDPE could be found closer to the surface, and the topography had less of an influence on the phase contrast. The AFM images obtained for strips containing 5 wt% LLDPE processed this way are presented in Figure 12. It can be seen in the phase images that there were differences in the material around the darker spots in the height images. Parallel alignment of the PP lamellae can be recognized in all four pictures, while the 2 × 2 µm close-ups show a clearer phase separation (Figure 12d). No structural difference between PP and LLDPE was recorded (Figure 12c). However, AFM is a surface analysis technique, and chemically similar materials are mainly discriminated by differences in their mechanical properties [26]. Therefore, the chemical composition of the darker spots were analyzed further by Raman spectrometry to gain a clearer understanding of the material composition throughout the strip.

**Figure 13:**
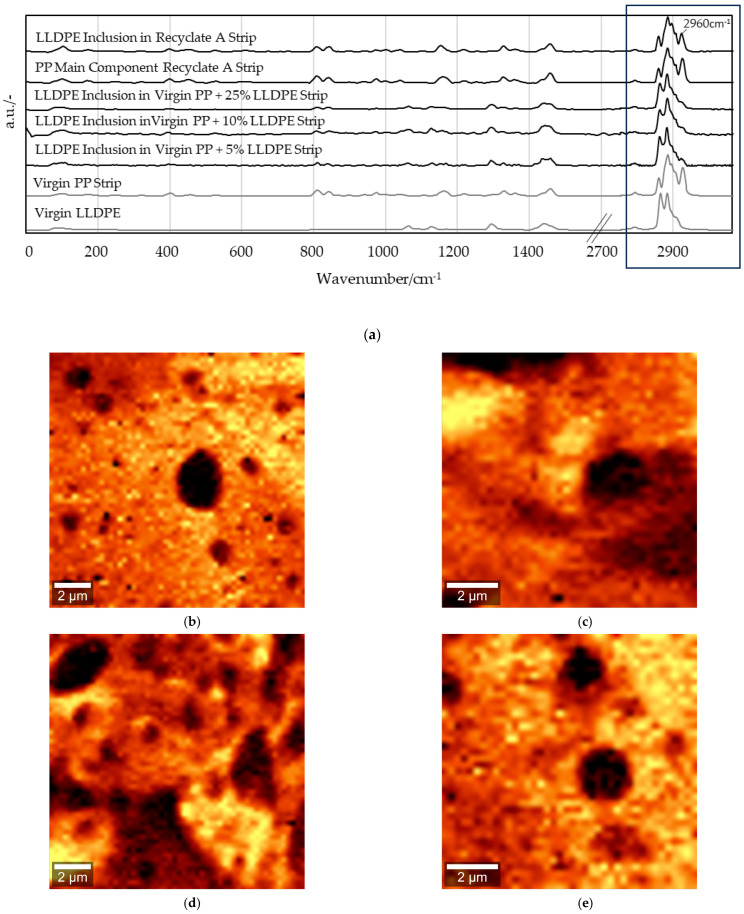
(**a**) Spectra of pure virgin PP and LLDPE, virgin PP-LLDPE blends (5–25 wt%) and pure recyclate A samples. Sum filter of the methyl side-group band at 2960 cm^−1^ (highlighted within the black frame) for 5 wt% (**b**), 10 wt% (**c**) and 25 wt% LLDPE in PP (**d**) and recyclate A (**e**).

Figure 13 shows 14 × 14 µm and 10 × 10 µm Raman spectroscopy image scans with 250 nm step-size measurements of 5 wt%, 10 wt% and 25 wt% blends of PP and LLDPE, which confirmed that the darker spot inclusions discovered by AFM consisted of LLDPE. The characteristic PP methyl side-group band at 2960 cm^−1^ began to fade when the scan reached the darker spot inclusion. PE inclusions smaller than 2 µm could be detected by the mixed PP/PE spectra [31]. In the case of 5 wt% LLDPE, hardly any PE was measured without also detecting the presence of PP. The resolution of a Raman spectrometer with a laser spot size of approximately 570 nm is insufficient to measure such small inclusions [31]. When the scan reached the LLDPE contaminations in recyclate A, the signal of the band at 2960 cm^−1^ became weaker [16].

### 3.3. Dynamic Mechanical Rheometric Testing of Blends and Recyclates and Assessment of LLDPE Present in PP

**Figure 14:**
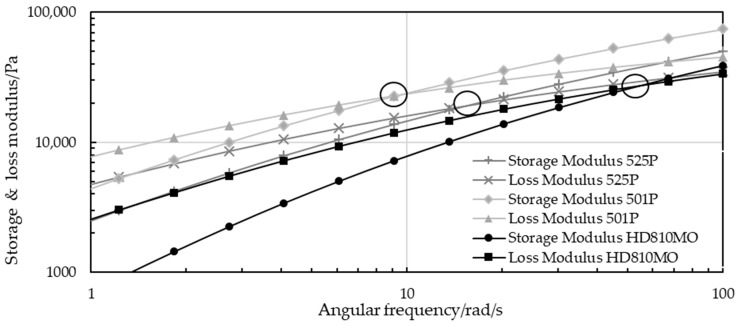
Exemplary results of three virgin material samples showing their cross-over points, highlighted by circles.

Figure 14 shows exemplary rheological measurements of three virgin PP strips. The evaluated cross-over points, highlighted with circles, display the shift from the primarily viscous to elastic domain.

**Figure 15:**
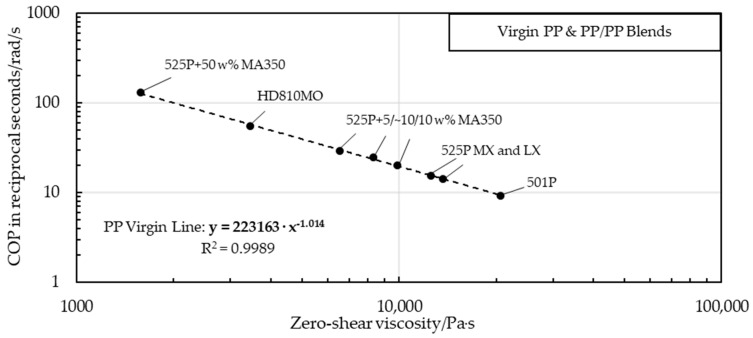
Correlation line and gradient of cross-over point over zero-shear viscosity for pure PP strips and PP-PP blends, and coefficient of determination.

Figure 15 shows the correlation between the zero-shear viscosity of multiple virgin PP samples with various zero-shear viscosities and the COP, which is defined as the intersection of the storage and loss moduli over the angular frequency. The zero-shear viscosity and the COP both depend entirely on the molar mass [32], provided that the material investigated is homogeneous [32,33]. An intersection at higher rads per second indicates a lower molecular mass, as the domain shifts from a predominantly viscous to dominantly solid phase require a higher angular testing frequency. A pronounced and clearly defined trend could be observed, primarily supported by the degree of determination of the double logarithmic gradient (Equation (3)).

(3) $$COP=223,163\;\cdot \;{\eta_{0}}^{-1.014}$$

**Figure 16:**
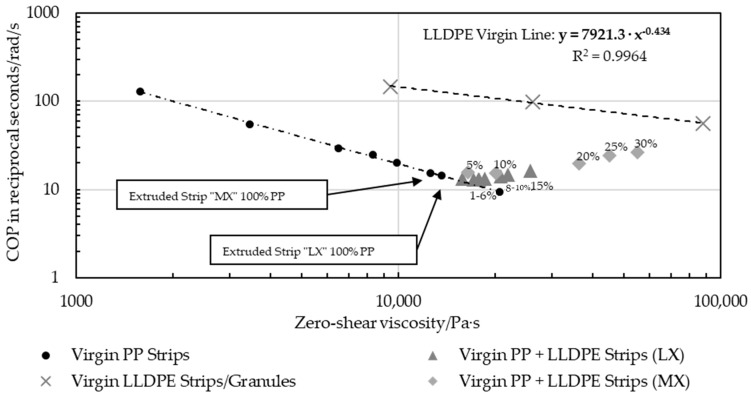
Double logarithmic trend of pure virgin PP and LLDPE samples, and MX strip and LX strip blends with 1 wt%–30 wt% LLDPE that diverged from the virgin PP gradient trend.

The presence of LLDPE in the PP disrupted the isotropy and caused a deviation from the PP correlation trend, depending on the amount of LLDPE. In Figure 16, virgin PP MX strips and LX strips with an increasing LLDPE content systematically deviated in the direction of the 100% LLDPE samples. This second dotted line resulted from the pure virgin LLDPE measured in a pure granule form, platelet, and MX extruded strip. Manually blended MX strips diverged slightly from the trend of the automatically blended LX strips.

**Figure 17:**
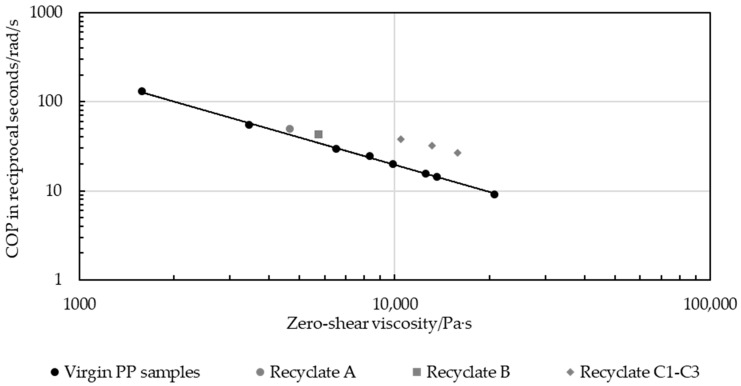
Deviations exhibited by recyclate samples A-C3 compared to the trend of pure virgin PP.

In Figure 17, all recyclates with an unknown PE content behaved similarly. No LLDPE was added to the recyclates in the experiments, as we expected significant quantities of PE to already be present. Rheometric measurements were thus used to quantify the LLDPE present.

## 4. Discussion

### 4.1. DSC Detection Hypothesis

The hypothesis that the LLDPE content can be approximated by DSC, especially at a low LLDPE content in PP, was refuted by the results for virgin blends of PP and LLDPE illustrated in Figure 4, Figure 5, Figure 6, Figure 7 and Figure 8. The results for strips produced with an automated dosing system for blending exhibited a trend that deviated significantly and systematically from those calculated based on the known LLDPE content. While sampling of these blends was in the milligrams range, which may not be representative, the consistent trend over all the samples demonstrated a divergence trend that cannot be interpreted as an accidental error. These enthalpy differences could be caused by the different evaluation methods, and by crosslinked LLDPE that obstructed the development of crystallinity, not only for its own domains but also for PP [4,34]. For recyclates B-C, but not for recyclate A, the presence of PE was shown qualitatively. PE inclusions in recyclate A were, however, confirmed by AFM. The validity of the quantitative results from standard DSC evaluation alone (linear or spline baseline integration) should be questioned.

### 4.2. AFM Detection

AFM can be used to analyze recrystallized samples, specifically, the arrangement of the polymeric chains. Virgin PP on its own exhibited large quantities of complete spherulites when it was allowed to crystallize from melt at 144 °C. The addition of LLDPE manifested in the topographic images as dark, round spots in these spherulites due to its contraction during crystallization at a lower temperature than PP, as well as a slight difference in the density, with LLDPE having a higher value. Since the isothermal step was within the temperature range for PP crystallization, it is possible that PP crystals partially covered the LLDPE domains during the process. For lower LLDPE quantities, the spot size and number were directly proportional to the amount of LLDPE present in the PP, while at 25 wt% LLDPE, the formation of crystalline PP structures was hindered. The same behavior was exhibited in recyclate A. The rough surface found for sample B could be due to the various additives usually added to the polymer during the recycling process.

In the samples cooled down without an isothermal step, the PP did not have enough time to rearrange into crystalline macrostructures (i.e., spherulites) first during this faster sample preparation, and LLDPE was found closer to the surface. This led to a distinction between the two polymers in the phase images, since the topography no longer had a strong influence on the contrast.

### 4.3. Raman Spectroscopy Detection

Raman spectroscopy proved to be an efficient method for qualitative chemical characterization of the PP samples and PE inclusions. As with DSC, the quantitative assessment of the complete sample composition by Raman spectroscopy is limited. Nonetheless, the excellent identification capabilities for small-scale inclusion (~2 µm) provides a deeper understanding of the LLDPE distributions in PP for fractions as low as 5 wt%. However, qualitative analysis of inclusions at the nanometer scale is not trivial, as inclusion can occur beyond the resolution limits of a confocal Raman spectrometer. A 532-nanometer laser results in a spot, requiring a narrow step size of 50 to 250 nanometers depending on the LLDPE inclusion size for the spectra to present a combined spectrum where a difference in the PP fingerprint intensity at 2960 cm^−1^ is measurable. This is displayed in the “image scan” sum filters and the spectra of the recyclate A sample. Still, the measurement and evaluation of low fractions with minuscule inclusions proved to be elaborate but valid in the MX strips investigated. The detection of some fillers in recyclates can pose a challenge when the substances that are present result in fluorescence, which occurred when measuring recyclates B and C1-C3. Here, a (near-) infrared laser could be used but will result in a larger laser spot size and lower resolution [16,31].

### 4.4. Determination of LLDPE in PP Using Rheometric Oscillatory Testing

The rheometric testing showed a correlation between the results from PP samples and a systematic deviation from this gradient that depended on the amount of PE present. Both MX-blended and LX-blended strips were manufactured by similar but not identical extrusion processes, so their offset points differed due to their molar masses, which were influenced by extrusion-related molecular degradation. For example, since the different die geometries (“MX” and “LX”) applied different shear stresses outside the nondestructive linear viscoelastic area [17], the correlation for the systematic deviations from the pure PP gradient that depend on LLDPE content cannot be exactly the same. In particular, PP strips blended by the automated system showed a coherent correlation and were therefore used to create the first rheometric model for calculating the LLDPE content. Even MX strips with potential fluctuations in the LLDPE content showed a similar trend.

**Figure 18:**
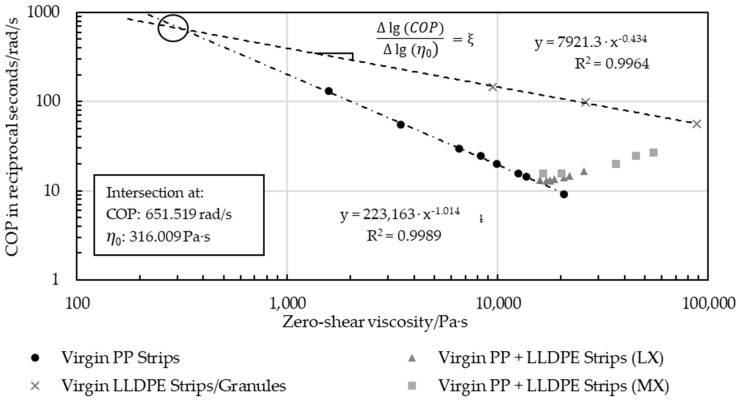
Intersection of pure virgin PP and LLDPE trend lines used to determine the slope (ξ) of the line that connects the COP over η0 of the intersection and to every COP over η0 point of all virgin blended samples.

**Figure 19:**
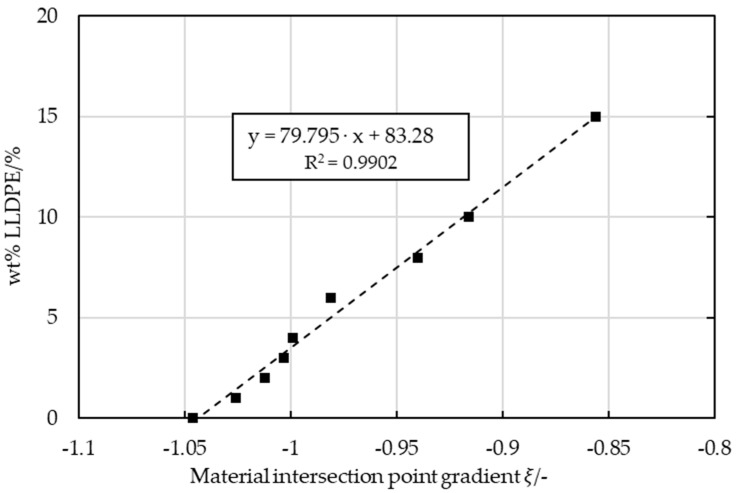
Slope (ξ) of the line connecting the material intersection to every virgin PP LX strip blended with an increasing LLDPE content.

The gradients of the pure PP samples and those of pure LLDPE (COP over *η*_0_) intersected at 651.519 rad/s and 316.009 Pa·s (Figure 18). Extrapolating a line from this intersection to every blend and recyclate yielded lines with various slopes that correlated with the LLDPE content, with the slope of pure PP being about −1 and that of pure LLDPE being about −0.43, as depicted in Figure 18.The slopes based on the intersection point and the COP over *η*_0_ of the LX virgin PP-LLDPE blends are displayed in Figure 19.

Samples containing a blend with a low LLDPE content (<15 wt%) showed a linear trend and the content can therefore be calculated using the following equation:

(4) wt% LLDPE in PP=79.795·ξ+83.28

With this model, the weight fractions of unknown LLDPE content in recyclates can be determined. Weight fractions for large proportions of LLDPE (>15 wt%) can be determined on the basis of MX strip samples. However, the overall LLDPE content of MX-extruded strips may have more variation.

**Figure 20:**
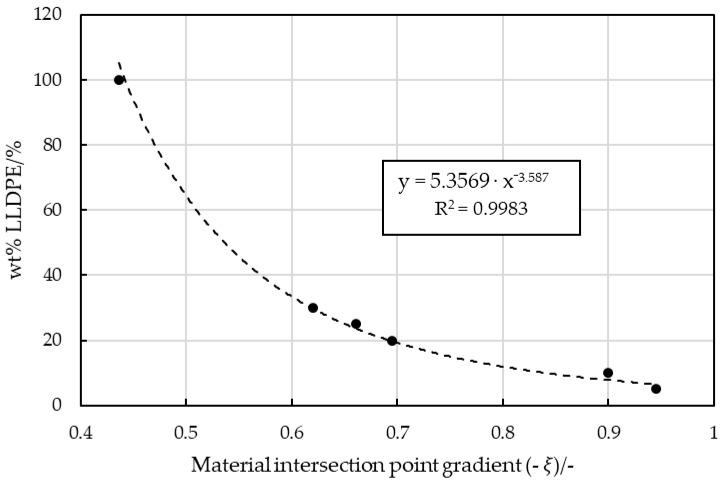
Slope (ξ) of the line connecting the material intersection to every virgin PP strip blended with an increasing LLDPE content.

Although the blended MX strips may have contained small unmeasured amounts of LLDPE, a correlating function could be identified (Figure 20). This power-law interpolation provides better predictions for higher LLDPE contents (>15 wt%):

(5) $$wt\%\;LLDPE\;in\;PP=5.3569\cdot (-1\cdot {\xi )}^{-3.587}.$$

With Equations (4) and (5), the PE/LLDPE content of the recyclates was determined, as listed in Table 6. The linear predictive estimation of the PE content in recyclate A is especially promising. Applying the linear approximation, a content of 1.24 wt% PE was suggested, which is plausible. Due to absence of a measurable melt enthalpy and insufficient inclusion size, neither DSC nor Raman spectroscopy was capable of detecting the presence of PE. LLDPE contamination was indicated only by the AFM results.

## 5. Conclusions

Using AFM and Raman spectroscopy, we gained insights into the distributive and dispersive characteristics of LLDPE contamination in PP. The determination of the overall LLDPE content, based on DSC melt and recrystallization enthalpies, showed that these methods are insufficiently precise to assess low proportions of contaminants (<15 wt%). Representative sampling using single-digit milligram specimens is often not feasible, as optimal mixing of the sample cannot be assumed. Additionally, the enthalpy determination using the mixed recrystallization peaks between LLDPE and PP varied too much, even in virgin material blends, to assess the weight percentages of LLDPE based on theoretically increasing recrystallization enthalpy. The enthalpy values obtained using spline baseline evaluation for the melting curves proved to be the most promising approach to estimate the actual value of LLDPE in PP since they are closest to the calculated enthalpy values. However, this method still required a distinguishable LLDPE melt peak. The melt and recrystallization enthalpies therefore deviated from the reference values because of numerous complications related to these evaluation methods and the physical properties of the polymer, most importantly, the LLDPE domain’s need for a critical crystallizable size to develop a standard crystalline morphology, as shown by AFM. Furthermore, depending on the degree to which it is crosslinked during processing, LLDPE inhibits crystallization, impeding the measurement of crystallinity.

We have presented an alternative analysis method that is based on rheometric oscillatory testing of PP melts contaminated with LLDPE. Crucial correlations between the zero-shear viscosity and COP that depend on the degree of polyolefin contamination were found. Since the correlation between the COP and zero-shear viscosity are not identical for every polymer, they are influenced disproportionally if cross-contaminations are present. Building on these correlations, the analyzed virgin LLDPE and PP and custom blends can be used to formulate two models for higher and lower levels of LLDPE contamination in PP. The appropriate model can be chosen based on the qualitative DSC results. The LLDPE content in recyclates can therefore be determined for up to 30 wt% contamination by using dynamic mechanical measurements with a compact modular rheometer and then applying the appropriate model to the outputs. Also, minimal contaminations around 1 wt% can be quantified, while DSC does not provide sufficient or any detection of the LLDPE content.

The effect of inorganic fillers or additives were neglected. Further investigations with other PE blend variants using precise mixing equipment and more samples for statistical substantiation will be necessary to corroborate the validity of the two mathematical models. Extending this approach to cover other forms of PE (e.g., HDPE and LDPE) and other polymers will require further examination and studies. These investigations opened up a novel characterization method especially when it comes to low wt% of cross-contaminations where other methods fail. Since there are more types of PE, this approach can be further built upon in further research. These types of PE contaminants in the post-consumer recyclates were not assessed in the scope of this paper with the methods used, but we expect them to follow the trend of LLDPE.

## Figures and Tables

**Table 4:** Pure and blended MX strip samples and analyses conducted, indicated by a checkmark.

Material/ Method	Fractions Added wt% PP|wt% LLDPE	Material Grade	Rheometry	DSC	Raman Spectroscopy	AFM
Virgin PP	100%|0%	501P	✔			
	100%|0%	525P	✔			
	100%|0%	HD810MO	✔			
	100%|0%	525P + 5 wt% MA350	✔			
	100%|0%	525P + 10 wt% MA350	✔			
	100%|0%	525P + 50 wt% MA350	✔			
Virgin PP + Virgin LLDPE	100%|0%	525P	✔	✔	✔	✔
	95%|5%	525P + Q1018H	✔	✔	✔	✔
	90%|10%	525P + Q1018H	✔	✔	✔	✔
	80%|20%	525P + Q1018H	✔	✔		✔
	75%|25%	525P + Q1018H	✔	✔	✔	✔
	70%|30%	525P + Q1018H	✔	✔		✔
Virgin LLDPE 1-3	100%|0%	Q1018H	✔	✔	✔	
Post-Consumer PP Recyclate A	100%|0%		✔	✔	✔	✔
Post-Consumer PP Recyclate B	100%|0%		✔	✔		✔
Post-Consumer PP Recyclate C 1–3	100%|0%		✔	✔		

**Table 5:** Pure and blended LX strip samples and measurements conducted, indicated by a checkmark.

Material/ Method	Fractions Added wt% PP|wt% LLDPE	Rheometry	AFM	Raman Spectroscopy	DSC
Virgin PP + LLDPE	100%|0%	✔			✔
	99%|1%	✔			✔
	98%|2%	✔			✔
	97%|3%	✔			✔
	96%|4%	✔			✔
	94%|6%	✔			✔
	92%|8%	✔			✔
	90%|10%	✔			✔
	85%|15%	✔			✔

**Table 6:** LLDPE content in PP calculated by two rheometric models.

Material/ Method	Fractions Added wt% PP|wt% LLDPE	ξ for Model Selection	Lin. Model: wt% (LLD)PE Content	P.-L-. Model: wt% (LLD)PE Content
Post-Consumer PP Recyclate A	100%|0%	−0.997	1.24	5.43
Post-Consumer PP Recyclate B	100%|0%	−0.976	3.05	5.86
Post-Consumer PP Recyclate C-1	100%|0%	−0.837	15.04	10.16
Post-Consumer PP Recyclate C-2	100%|0%	−0.835	15.21	10.25
Post-Consumer PP Recyclate C-3	100%|0%	−0.831	15.56	10.43

## Data Availability

The raw data supporting the conclusions of this article will be made available by the authors on request.
